# Uncovering Art’s Vanishing Hues with Surface-Enhanced Raman Scattering: Drawing Inspiration from the Past for the Future

**DOI:** 10.1021/acsnano.4c05389

**Published:** 2024-06-26

**Authors:** Kristin L. Wustholz, Shelley A. Svoboda, Meredith G. Martin, Benjamin T. Steinman, Zhaoyun Zheng

**Affiliations:** † Department of Chemistry, William & Mary, Williamsburg, Virginia 23187, United States; ‡ Paintings Conservation, Colonial Williamsburg Foundation, Williamsburg, Virginia 23187, United States

**Keywords:** Surface-enhanced Raman scattering (SERS) spectroscopy, plasmonics, nanoparticles, cultural heritage, art conservation, dyes, pigments, fading

## Abstract

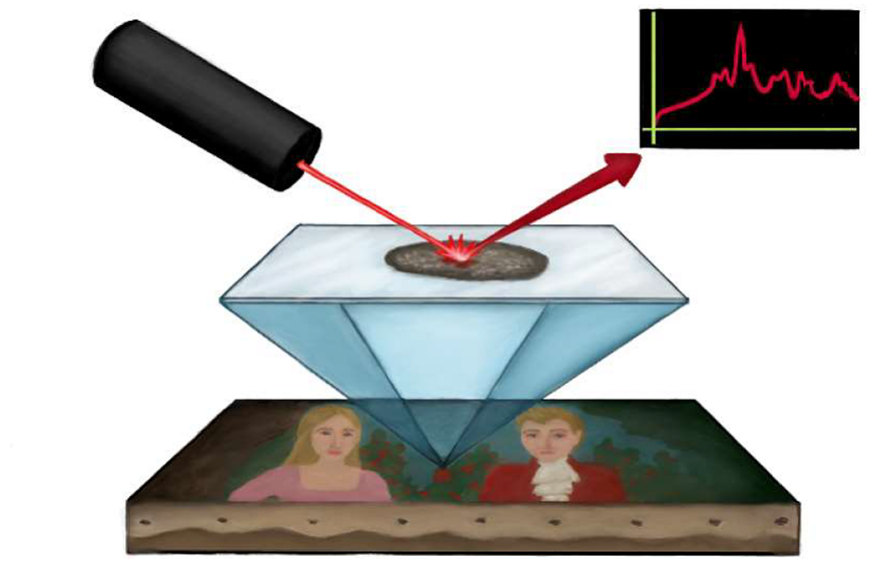

The aesthetic and historical significance of art is well recognized; art can stoke emotions, invite close inquiry, and connect us to the past. However, works of art are also complex material objects that present unique challenges and opportunities for the scientific community. Identifying “fugitive” organic pigments in traditional oil paintings, for example, presents a particularly complex analytical challenge that is critical to address for their conservation and long-term preservation. In this Perspective, we discuss the benefits and technical challenges of applying surface-enhanced Raman scattering (SERS) spectroscopy to the ultrasensitive identification of fugitive pigments in paintings as well as future developments in SERS we envision that are inspired by the past.

As complex material objects, works of art and the corresponding challenges they present to museum professionals provide rich fodder for scientific innovation.^1^ For example, the identification of “fugitive” or fading organic chromophores in paintings is an important, although technically difficult, endeavor. The successful characterization of organic dyes and pigments contributes to understanding artists’ material choices and original intent, provenance and attribution studies, conservation treatments, and long-term preservation strategies. However, identifying these colorants in paintings is extremely difficult. Due to their high tinting strength (i.e., extinction coefficients) and propensity to fade, organic dyes and pigments are usually present in extremely low concentrations within a complex paint matrix that contains interfering colorants and binding media. The natural aging and degradation processes, as well as previous restorations that take place over time, can further complicate analysis. Furthermore, because works of art are irreplaceable, sampling is extremely limited—often to samples less than ∼100 μm in diameter—or not possible. Due to its exquisite sensitivity and selectivity, especially for organic chromophores, surface-enhanced Raman scattering (SERS) spectroscopy has emerged as a powerful solution to these problems.^2−5^ In this Perspective, we highlight a set of colorful case studies resulting from close collaboration between our institutions, where SERS has offered insights about the artist’s palette and pigment fading in paint. Alongside its benefits, we also discuss the technical challenges inherent in SERS-based painting analyses, as well as how we envision that the past can inspire future advancements.

## Why SERS Shines for Painting Analysis

Paintings are typically made of a complex layering of materials that is exceedingly heterogeneous. Traditional oil paintings, for example, contain multiple layers of water-insoluble pigment mixtures possessing varied chemical and physical properties, that are bound in protein-, gum-, or oil-based media. This inherent complexity, combined with low analyte concentrations, light-induced fading, and micrometer-scale sample restrictions, makes the identification of organic dyes and pigments in paintings a massive analytical challenge, akin to finding a needle in a haystack. Indeed, the techniques routinely used in inorganic pigment analysis are not appropriate for organic dye-based materials.^5^ X-ray fluorescence (XRF) and scanning electron microscopy with energy-dispersive X-ray spectroscopy (SEM-EDS/X) lack elemental signatures for organic dyes. However, these methods can probe which metal cation is bound to the dye to form an insoluble pigment suitable for painting (e.g., aluminum from the common mordant “alum,” hydrated KAl(SO_4_)_2_). Traditional vibrational techniques (i.e., Fourier transform infrared and Raman spectroscopies) are unsuitable due to interference from binders and extenders or dye fluorescence, respectively. Although noninvasive, UV/vis reflectance spectroscopy, UV fluorescence imaging, and multispectral or hyperspectral imaging yield broad and overlapping signals that vary with the surrounding environment and cannot unambiguously identify organic dyes and pigments. High-performance liquid chromatography (HPLC) is useful for larger samples from cultural heritage objects such as millimeter-sized threads from dyed textiles, but samples from paintings and other polychrome works of art are generally too small (i.e., micrometer-scale in diameter) and/or dye concentrations are too low. SERS offers an elegant alternative to these approaches.

In SERS, a nanostructured noble-metal substrate provides benefits that are 2-fold: extremely enhanced Raman signals such that microscopic samples are identifiable as well as quenching of the interfering fluorescence produced by many organic colorants.^3,6^ SERS signals can be further enhanced to factors of ∼10^8^ or more relative to normal Raman scattering—down to the single-molecule limit—when the exciting field is resonant with a chromophore analyte.^7^ SERS is thus ideally suited for the ultrasensitive detection and identification of organic colorants. Indeed, ten years after the discovery of surface-enhanced Raman signals from pyridine on electrochemically roughened Ag electrodes, the SERS-based identification of a fugitive organic pigment in a historical dyed textile was reported.^8^ Years later, following several advances in nanofabrication, instrumentation, and mechanistic understanding as well as the creative and pioneering collaborations between several museum professionals and chemists, SERS studies of art objects began to experience a renaissance. By 2011, SERS had proven to be an effective method to identify organic dyes and pigments in textiles, watercolors, pastels, and even paint glazes.^4,5,9−15^

We became interested in the SERS-based identification of organic dyes and pigments in historic oil paintings from the Colonial Williamsburg Foundation (CWF) collection, both to advance SERS technology and local conservation efforts as well as to provide integrated research experiences for students.^16,17^ For example, the seminal SERS investigations of art objects were limited to a relatively small number of red organic chromophores (e.g., carmine and madder lake pigments and their associated water-soluble dyes carminic acid, alizarin, purpurin, etc.) and/or used HF to pretreat glaze samples for analysis. For SERS to reach its full potential in the museum setting, we initially sought to identify a broad palette of organic color-bodies in paintings and avoid harsh pretreatments, which irreversibly alter the sample and are sometimes deemed unsuitable for the conservation or undergraduate laboratory setting. Our strategy has been to allow these historic oil paintings, along with the unique challenges and questions they present, to inspire our research efforts for over a decade. Doing so has yielded a powerful SERS toolbox capable of identifying a wide range of organic and inorganic pigments in actual works of art.

## Revealing Fugitive Red Pigments in Oil Paintings

Due to the complexity, uniqueness, and extremely small size of individual paint samples, translating the promise of SERS into real-world practice has required overcoming several technical challenges. Perhaps the most critical aspect of SERS is to ensure that analytes adsorb to the surface of the nanostructured metal substrate to experience the enhanced electromagnetic fields (i.e., electromagnetic enhancement mechanism) that generate signal amplification and concomitant fluorescence quenching.^6,18^ That is, both the SERS-active substrate and the analyte affinity (e.g., solubility, electrostatics, and interference from other molecules in the sample) must be carefully considered. Countless studies have been dedicated to the synthesis and fabrication of various nanostructured substrates for SERS, representing a wide variety of materials, geometries, sizes, and in the case of suspended colloidal nanoparticles, capping agents.^3^ The efficacy of various SERS substrates for art and archeological samples has also been assessed.^10,13^ We generally employ colloidal suspensions of Ag nanoparticles prepared via the Lee and Meisel method,^19,20^ where citrate acts as both reducing and capping agents. This straightforward synthetic preparation uses low-cost materials to generate a polydisperse suspension of nanoparticles that exhibits a broad plasmon resonance suitable for various colorants and exciting lasers in the visible region. Moreover, citrate-reduced Ag colloids are well-known for their ability, once aggregated, to detect individual molecules.^7,21,22^

**Figure 1:**
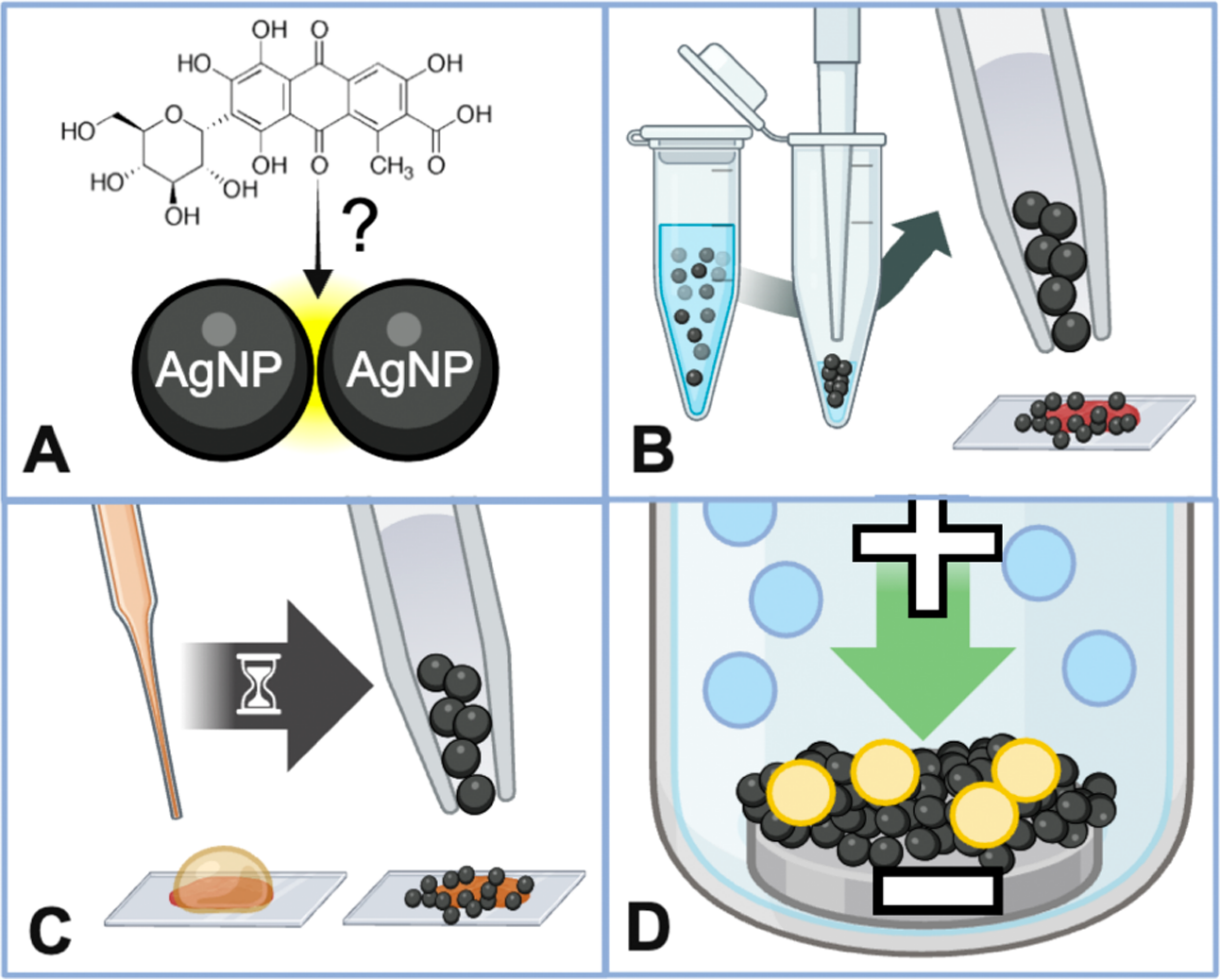
(A) SERS methodologies for fugitive organic pigment detection in paintings, which modify the mechanism to deliver the dye analyte to the surface of the AgNP enhancing substrate. (B) “Direct” or extractionless nonhydrolysis SERS method. AgNPs are centrifuged into a colloidal paste to aggregate particles and remove excess citrate and then applied directly to microscopic dye, pigment, or paint sample. (C) Many pigment and paint samples benefit from pretreatment to hydrolyze and/or extract soluble dyes prior to applying the AgNP paste for SERS measurements. In addition to variation of the treatment type and materials used (e.g., solvents for extraction or acids for hydrolysis), the treatment time and temperature can be adjusted to further optimize dye removal for SERS. (D) EC-SERS method to identify the dyes extracted from pigments by using AgNP-modified electrodes. Adjusting the applied voltage results in the displacement of blue interfering species and preferential surface adsorption of yellow dyes to the SERS-active substrate.

Apart from the substrate, it is also essential to consider how the analyte(s) will be delivered to the surface of the SERS substrate for detection. The first necessary step is sampling the artwork, which typically involves careful extraction of a microscopic particle by a conservator under a microscope. Gel microextraction is another minimally invasive strategy to sample paint for SERS.^23^ After sampling, the analyte must be brought to the noble metal surface and displace adsorbed citrate (see Figure 1A), a particularly challenging task for so-called “organic lake pigments”, insoluble particles comprised of dyes bound to a metal ion such as calcium or aluminum, which exhibit poor affinity for SERS substrates. Therefore, the early SERS studies of art utilized sample pretreatment—extraction and/or hydrolysis steps in various solvents, acids, and alkali—to release the dye from its mordant and facilitate surface adsorption.^9,10,14^ However, these steps not only require some prior knowledge of the sample but also forever alter its chemistry and morphology. We wondered if aggregated citrate-reduced Ag colloids (AgNPs) could be used to identify fugitive organic pigments in oil paintings—without sample pretreatment.

**Figure 2:**
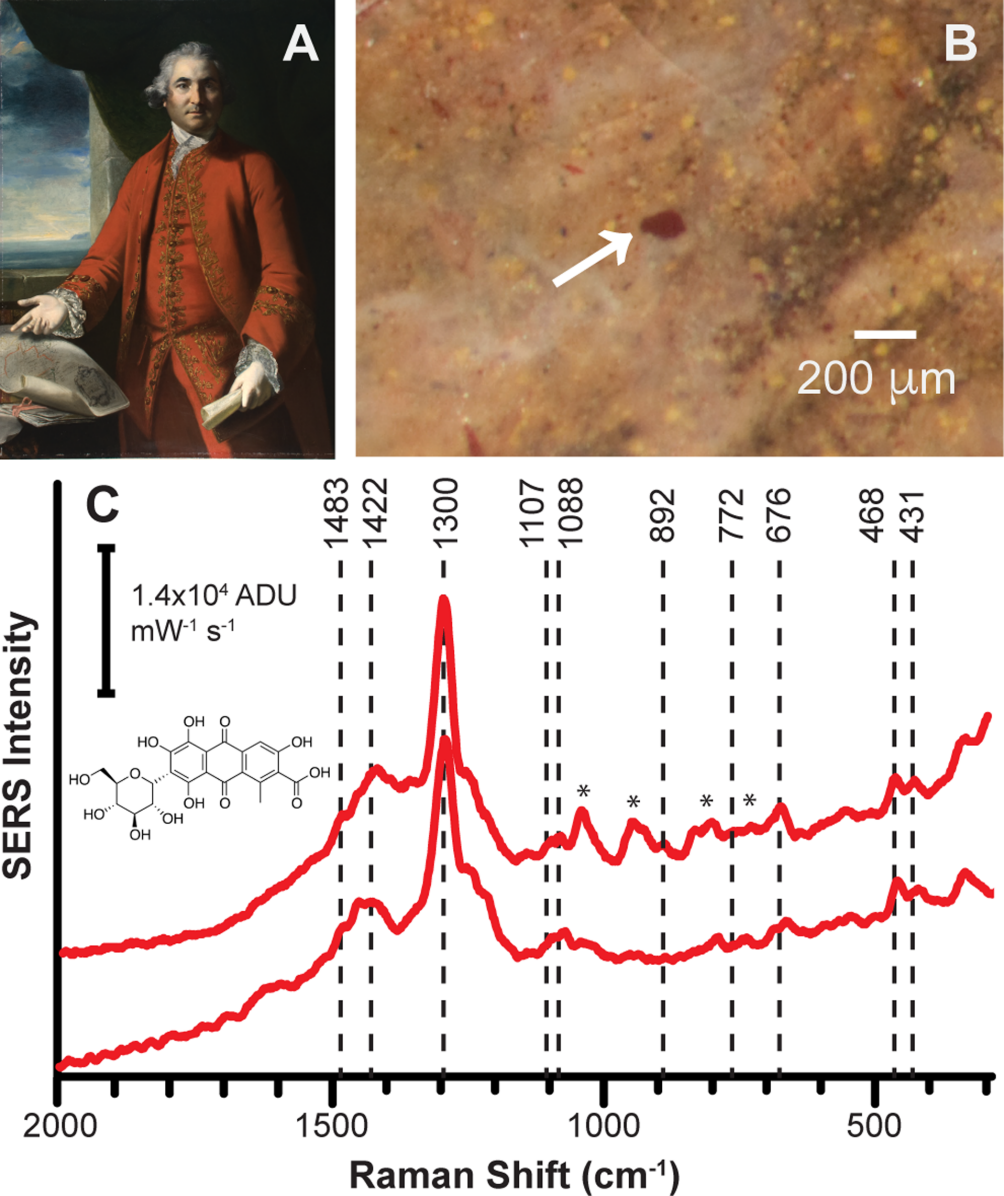
SERS study of red lake pigments in (A) *Portrait of Isaac Barré* (1766) by Sir Joshua Reynolds. (B) A surface photomicrograph of the sitter’s cheek shows a red lake pigment within a wide variety of hues and particle shapes, consistent with tremendous material complexity. (C) Extractionless nonhydrolysis SERS spectra of a (top) dispersed sample from sitter’s cheek and (bottom) cross-section sample from sitter’s coat obtained at 632.8 nm. Labeled peaks are consistent with carmine lake, and asterisks denote interfering peaks due to adsorbed citrate. [Adapted from ref (24). Copyright 2011, American Chemical Society, Washington, DC.]

Our early SERS studies were inspired by two paintings in the CWF collection: *Portrait of William Nelson* (probably 1748–1750) by Robert Feke, one of the earliest known artists born in the American colonies, and *Portrait of Isaac Barré* (1766) by Sir Joshua Reynolds, cofounder of the Royal Academy of Arts in London. In both paintings, portions of the sitter’s fleshtones appear slightly faded, suggesting the presence of fugitive red lake pigments. To investigate further, we implemented an approach developed by Brosseau et al. to aggregate AgNPs via centrifugation, thereby generating electromagnetic hot spots to enhance SERS signals, and then to directly apply the resulting colloidal “paste” to an unknown sample (Figure 1B).^12,13^ Despite the small size (i.e., ∼20–50 μm in diameter) and exceptional material complexity of these paint samples, “direct” or extractionless nonhydrolysis SERS (i.e., without any pretreatment) provided definitive identification of carmine lake pigment in the flesh regions of both paintings (Figure 2).^24^ These findings demonstrated that an exceptionally small pigment particle—also called a dispersed sample—is adequate to produce high-quality SERS spectra of organic pigments in oil paint, and without the need for sample pretreatment. Furthermore, the finding of carmine lake in both portraits informs museum curators and conservators about the artists’ material choices, pigment availability, as well as fading. Encouraged by these results, we next attempted extractionless nonhydrolysis SERS measurements of other types of samples and organic pigments likely to be found in the museum’s collection of paintings.

Cross-section sampling is a microdestructive technique that is commonly employed in the conservation setting to image the various layers (e.g., ground, paint, varnish) within paintings or painted objects. Although cross sections are larger than dispersed samples (i.e., approximately millimeter-scale), they are sufficiently small to not visibly alter artworks and have the benefit of revealing a broader scope of information about an artist’s technique, palette, as well as paint condition. After cross-section samples are mounted in a transparent resin, their layered structure can be visualized using various techniques that include visible, UV, and polarized light microscopy as well as SEM-EDX. Although these techniques serve to visualize the layers and help characterize pigments, they cannot unambiguously identify any organic colorants embedded within.

We investigated mounted cross-section samples obtained from *Portrait of Isaac Barré*, *Portrait of Elizabeth Burwell Nelson* by Robert Feke, as well as dispersed samples from *Young Woman in a Red Dress* (1890) by Gabriel de Cool using SERS.^25^ In all three paintings, cross-section samples could not be taken from the sitters’ fleshtone regions, which highlights the unique power of SERS to identify fugitive pigment grains within small but important areas of paintings. However, a cross-section sample from the sitter’s coat in *Portrait of Isaac Barré* could be obtained and showed the presence of a preparation layer, a paint layer containing an inorganic red colorant, and a glaze layer containing a red lake pigment as evidenced by fluorescence under UV illumination. We applied centrifuged AgNPs directly to this mounted cross-section and performed SERS measurements, which revealed a carmine lake in the glaze applied over a layer rich in the inorganic pigment vermilion. This combination of SERS with traditional cross-section analysis informed on Reynolds’ material choices and technique, particularly his preference for carmine, despite disparaging it as a “treacherous” colorant due to its fading.^26^ Using the same approach, SERS analysis identified carmine lake in a cross-section sample taken from the rose bud held by the sitter in Robert Feke’s painting as well as madder lake in reference cross sections and dispersed samples from *Young Woman in a Red Dress*. Importantly, this study demonstrated that AgNPs could be directly applied to (and later removed from) cross-section samples for SERS analysis of red lake pigments, greatly expanding the scope of this approach to include the multitude of cross-section samples that have been (and continue to be) obtained from paintings and stored in collections around the world.^25^

Collectively, these early studies showed that extractionless nonhydrolysis SERS is extremely effective for the identification of fugitive red pigments in oil paintings. Indeed, the identification of carmine lake, madder lake, and their water-soluble dye constituents continues to find the broadest application in SERS-based studies of cultural heritage objects. Although red lakes were prized by painters due to their hues, tinting strengths, and translucency, many other organic colorants are likely to have found use in traditional oil paintings and contribute to fading. Unfortunately, we soon discovered that yellow and blue organic-based pigments could not be reliably identified using SERS—even in relatively large reference samples. Modest SERS signals have been attributed to poor surface affinity, solubility, and/or relatively small resonance Raman enhancements.^6,22,27^ To uncover the identity of these colorants and expand the applicability of SERS beyond fugitive red pigments, we began to investigate reproducible strategies to enhance the surface affinity of these analytes for the SERS substrate.

## SERS Advances around the Color Wheel

Analytes must be within a few nanometers of the noble metal substrate, and preferably in electromagnetic hot spots, to experience the full power of SERS.^28^ Since fugitive pigments in oil paintings are insoluble particles embedded within complex oil-, wax-, or protein-rich matrices, many studies utilize sample pretreatment to liberate the dye from its surroundings and promote surface adsorption to the SERS substrate (Figure 1C). For example, Leona at the Metropolitan Museum of Art pioneered the use of HF vapor to hydrolyze the dye-metal bond in organic lake pigments to provide for SERS detection.^14^ Our first foray into paint sample pretreatment was inspired by the *Portrait of Evelyn Byrd* (probably 1725–1726). Initial examination of the painting in the conservation laboratory revealed apparent fading of the sitter’s blue dress, indicating an organic-based pigment such as indigo. To definitively identify the colorant responsible for photofading, we attempted SERS measurements on various blue reference pigments with and without sample pretreatment, although HF was not used for safety reasons. In all cases, even when using salts and acids (e.g., NaCl, HCl) to modify the structure and surface chemistry of AgNPs as well as pH and indigo solubility as previously reported,^29−31^ high-quality SERS spectra of indigo could not be obtained.

Unlike lake pigments, indigo is insoluble due to intramolecular hydrogen bonding, which precludes its solvation in water and likely limits its interaction with the aqueous suspension of AgNPs. One art-inspired strategy to enhance surface adsorption is to convert indigo to its soluble form, *leuco* indigo, a pretreatment approach inspired by the vat dyeing process used for millennia to create indigo-dyed textiles. However, *leuco* indigo is colorless, which eliminates signal enhancement through a resonance Raman effect. We developed an alternative pretreatment strategy based on the *in situ* conversion of indigo to form another blue colorant, soluble indigo carmine, using microliter quantities of H_2_SO_4_.^32^ Using this approach, we were surprised to discover that dispersed samples from the sitter’s dress in *Portrait of Evelyn Byrd* contain both indigo and Prussian blue. These findings represented an early example of a merchant or artist mixing indigo with inexpensive Prussian blue, as well as informed digital restoration of the painting to share with the public on museum exhibition labels.

This study also laid the groundwork for our growing interest in identifying pigment mixtures within a single paint sample using SERS. Since chromophore detection using SERS is dependent on multiple factors, including the optical properties of the enhancing substrate, surface affinity of the analyte(s), surface coverage, and the extent of resonance Raman enhancement, identifying pigment mixtures represents a major hurdle. We set out to address an important and well-known problem in conservation: the fading of paint that contains yellow organic colorants. For example, artists and suppliers frequently prepared optical mixtures of blue and yellow organic pigments to obtain green hues in oil paintings. Unfortunately, since yellow organic pigments derived from various plant sources (e.g., Reseda lake from weld, Stil de Grain lake from Buckthorn berries)—predominately flavonoid derivatives—undergo severe photofading upon exposure to light over time, much of these once-green regions of paintings now appear blue. Although the chemical and physical properties of artists’ blue pigments differ significantly from those of yellow organic pigments, we sought to devise a general strategy to reveal both color bodies within a single microscopic paint sample using SERS.

Several studies have detected fugitive yellow pigments in dyed textiles either with or without sample pretreatment.^33−35^ However, their identification in microscopic, aged paint samples has proven far more challenging due to severe fading, which can render samples invisible, as well as sample size restrictions and the sheer chemical complexity of yellow pigments obtained from natural sources. In 2013, we systematically evaluated several pretreatment strategies, used to hydrolyze and/or extract dyes from lakes as well as alter the surface chemistry of the nanoparticles, for SERS-based detection of various yellow organic colorants in historic oil paintings.^36^ Ultimately, a simple pretreatment strategy based on a 1:3 mixture of 1 M HCl in methanol produced high-quality SERS spectra of all tested yellow dyes, lake pigments, and reference paint samples, with the notable exception of gamboge resin, which required extraction in acetonitrile. With a simple methodology in hand to identify unknown yellow organic pigments in paint, we turned back to the CWF collection to address questions related to photofading and color shifts from green to blue.

Our previous SERS study of *Portrait of Elizabeth Burwell Nelson* identified carmine lake in the rose held in the sitter’s hand. Cross-section analysis of the corresponding rose stem, which now appears blue instead of green, revealed a blue and yellow pigment mixture possessing a broad range of particle sizes. Furthermore, the cross-section showed the absence of yellow pigments at the upper surface, consistent with light-induced fading. To identify both pigments in this region, we examined a dispersed sample from the stem using a SERS treatment flowchart approach, which integrates various treatments (e.g., H_2_SO_4_ for indigo and Prussian blue, HCl and methanol for yellow organics) into a stepwise procedure.^37^ By execution of this treatment flowchart approach, SERS measurements revealed the presence of Reseda lake and Prussian blue, two pigments with different hues and properties, within a single paint sample.

These studies and others provide convincing evidence that sample pretreatment is a valuable, even necessary, step prior to SERS analysis.^38^ Indeed, over the years, we have noted that ∼10%–20% of red lake samples from works of art cannot be identified using direct SERS, presumably due to poor interactions between the analyte and substrate. Our recent examination of several early American portraits also revealed the presence of red textile fibers with submillimeter dimensions in paint, some of which could also not be identified.^39^ To develop a more reliable SERS-based identification for micrometer-scale red lake pigments and fibers, the latter of which may derive from textiles used in the original pigment manufacturing process, we revisited the red lakes and their pretreatment using various solvents, acids, concentrations, and times. Importantly, the characteristic SERS peaks for carmine lake (i.e., at ∼1418, 1323, 1297, 474, and 434 cm^–1^) are routinely observed with or without pretreatment, but micrometer-scale madder-containing samples show significantly better SERS signals after extraction and hydrolysis. The optimal SERS results for carmine and madder lakes, in terms of signal intensities and peak separation, are observed for samples subjected to microliter aliquots of 1:2 solutions of 1 M HCl in methanol for at least 1 h prior to the addition of AgNPs. Shorter treatment times yield an overwhelming SERS signal at ∼240 cm^–1^ due to adsorbed chloride (i.e., AgCl), instead of the analyte of interest.

**Figure 3:**
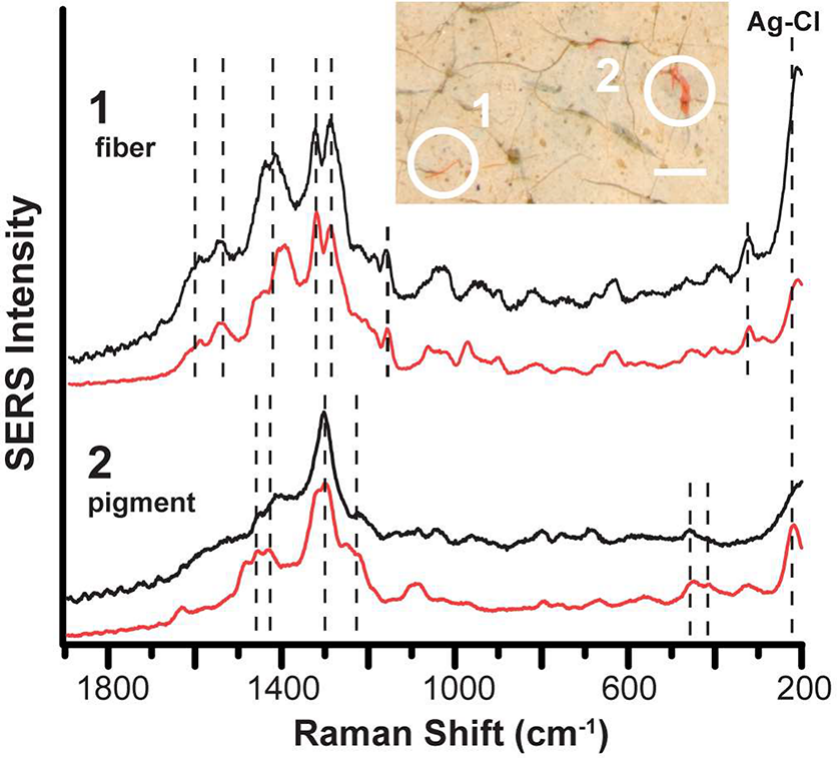
Pretreatment SERS of unknown red colorants in a Joshua Johnson portrait. Surface photomicrograph of the sitter’s flesh shows minute quantities of red colorants with a (1) fiberlike or (2) crystalline appearance. Scale bar = 100 μm. (Black) SERS spectra of unknown samples 1 and 2, obtained using a 632.8 nm laser at ∼10 μW, are excellent matches to (red) reference spectra of madder lake and carmine lake, respectively. Vertical dashed lines mark characteristic peaks for carmine lake, madder lake, or AgCl.

Figure 3 presents a case study where this pretreatment approach selectively reveals the identities of two different red organic colorants within the same region of *Portrait of Susanna Cardwell McCausland (Mrs. James McCausland) and Child* (ca. 1805) by Joshua Johnson. Although Johnson is an important figure and one of the earliest professional Black painters in America, little is reported about his painting materials and techniques. A surface photomicrograph of the sitter’s flesh reveals minute quantities of what appear to be red fibers as well as pink crystalline pigments. Pretreatment of these microscopic fiber and pigment samples with 1:2 HCl:methanol for ∼2 h yielded excellent SERS results. The fiber sample exhibits SERS peaks at 1600, 1548, 1449, 1414, 1326, 1296, 1232, 1192, 1162, 644, 564, 467, 397, and 320 cm^–1^, consistent with reference madder lake. Corresponding SERS spectra of the nearby crystalline pigment, however, are an excellent match to those of the carmine lake (i.e., characteristic peaks at 1457, 1422, 1304, 459, and 422 cm^–1^). By implementing this sample pretreatment approach, SERS identifies two distinct analytes associated with two very different physical substrates, despite their extremely low concentrations in the real-world environment of an aged paint layer. The findings of a madder-containing fiber and a carmine lake pigment provide insights into Johnson’s material choices and pigment sources as well as a better understanding of the appearance and future exhibition needs of this important painting.

## Art Lessons: Challenges and Opportunities

Questions of paint fading and artists’ choices have guided much of our work in SERS. In doing so, we and others have shown that SERS can find the elusive needle in a haystack—revealing a wide variety of natural and synthetic fugitive organic dyes and pigments in miniscule samples from paintings, even within samples that are spatially and chemically complex. Furthermore, the AgNP-decorated dispersed and cross-section samples that have been stored in our laboratory for many years—in some cases, for over a decade—continue to produce high-quality SERS spectra. Despite these advances in SERS technology and local conservation efforts, the goal of understanding and protecting paintings continues to provide challenges and opportunities for the future.

Every painting is inherently unique, possessing chemical and physical properties with potentially infinite complexities. The dispersed samples from these paintings, obtained using a surgical scalpel under a microscope, can be embedded in various hard-to-remove binding media or varnishes, contain both modern and aged restoration materials, and exhibit multiple hues. Even in rare cases where a single, bare pigment grain appears to be extracted, the variations in lake pigment chemistry and morphology mean that SERS-based identification is not guaranteed. In our estimation, based on analyzing hundreds of samples over many years, ∼85% of dispersed paint samples that are thought to contain red lake pigments can be readily identified using direct SERS, a success rate that can be improved to near unity with sample pretreatments (see, e.g., Figure 3).^2,4,38,40^ We have also shown that carmine lake is easier to identify than madder lake using AgNPs, even with pretreatment, a surprising result given that alizarin, the main chromophore in madder, exhibits exceptionally high-quality spectra and is frequently used to benchmark SERS substrates and methods. The origins of madder’s elusiveness are unclear, complicated by our limited ability to elucidate the crystal structures and corresponding photophysical properties of lake pigments. The detection of fugitive yellow organic colorants also continues to be challenging, most likely due to severe fading, which means that virtually no chromophores remain at the upper paint surface as well as the yellowing of binders that obscures their visibility. Collectively, these difficulties and others mean that the evolution of SERS into a broad-application method for fugitive pigment identification in paintings has remained elusive.

Several advances, some of which are inspired by seminal SERS experiments, have tremendous potential to broaden the application of SERS in the conservation setting. From our perspective, the sheer complexity of paint samples warrants significant effort focused on the integration of SERS with a separation technique. Although we successfully integrated three sample pretreatments into a flowchart method to identify blue and yellow organic pigments using SERS^37^—effectively handling one pigment at a time—the true heterogeneity of aged paint requires a method capable of handling many more analytes and interfering components. Several groups have shown that the combination of SERS with chromatography is well-suited to address the chemical complexity of artists’ materials. Brosseau et al. reported the integration of SERS with thin layer chromatography (TLC) to discern various dye components in reference pigments and dyed textiles.^13^ Although TLC-SERS and HPLC-SERS^41^ are useful to separate and identify dyes in a complex mixture, the latter being especially useful for closely related dyes from biological sources, these approaches require large samples that currently preclude their application to paintings.

Perhaps it is fitting, near the 50th anniversary of Fleischmann’s report of pyridine on electrochemically roughened electrodes,^42^ which, along with seminal works by Creighton^43^ and Van Duyne,^44^ is considered to have started the field of SERS, that we highlight the use of electrochemistry to address the ongoing challenges involved with the SERS-based detection of artists’ pigments. Electrochemistry is not typically regarded as a separation technique, but in electrochemical SERS (EC-SERS), applying a voltage to the SERS substrate in the presence of an electrolyte can lead to preferential surface adsorption and corresponding signal enhancements of analytes in sequential fashion (Figure 1D).^27^ Adjusting the applied voltage can enhance EC-SERS signals through electrostatic interactions (i.e., the surface charge of Ag becomes less positive as the potential is stepped in the negative direction), desorption of interfering species, charge transfer (i.e., the chemical enhancement mechanism), and, in some cases, potential-induced analyte reorientation. For example, Bindesri et al. demonstrated that although cannabinoid derivatives are not detected using traditional SERS with AgNPs, strong EC-SERS signals for tetrahydrocannabinol and carboxy-tetrahydrocannabinol are observed at different voltages on AgNP-coated electrodes (i.e., −0.4 V and −0.8 V, respectively), even at low concentrations and within bodily fluids that contain several competing species.^45^ Inspired by the enhanced selectivity and sensitivity of EC-SERS, we investigated its ability to separate and identify dyes in yellow lake pigments.

For this collaborative study,^46^ low-cost screen-printed electrodes modified with AgNPs are treated with chloride salt to displace adsorbed citrate, which is known to interfere both physically and spectrally (see, e.g., Figure 2). Next, we tuned the applied voltage to remove chloride from the enhancing substrate and then amplify the EC-SERS signals of eight yellow reference dyes via increased surface adsorption (e.g., signals for the closely related dyes quercetin and rhamnetin are maximized at −0.3 and 0.0 V, respectively). To identify the yellow dye components of lake pigments using EC-SERS, we treated microscopic samples of Reseda and Stil de Grain lake pigments with HCl and methanol to hydrolyze and extract the embedded dyes. Corresponding EC-SERS measurements of pigment extracts revealed high-quality, reproducible SERS spectra of the dye components, as well as greatly enhanced signals, compared to traditional SERS. Thus, performing SERS under electrochemical control yields definitive signals from weakly adsorbing analytes, even when present in a complex mixture, such as a lake pigment. This study and others^47^ highlight the potential for EC-SERS to address many persistent questions about artists’ materials—including the identification of fugitive yellow pigments and their dye constituents within microscopic dispersed or cross-section samples from paintings and other polychrome works of art.

In addition to EC-SERS, we anticipate that advances in machine learning for spectral analysis and classification will further expand the capabilities of SERS^48,49^ and other tools in nanoscience to increasingly complex samples and problems in conservation. For example, we recently demonstrated that several artists’ colorants (i.e., rhodamine and anthraquinone dyes) can be classified with machine learning—down to the single-molecule detection limit—using their intrinsic fluorescence dynamics.^50^ We envision that research at the intersection of nanoscience and machine learning, which targets cultural heritage objects with extreme degradation and sampling challenges, will continue to generate knowledge and pathways that are currently unseen.

## Dedication

We are grateful to the creative scientists, museum professionals, and donors who have contributed to research at the interface of SERS and art over the years, especially the late Prof. Richard P. Van Duyne—a stellar scientist, mentor, and human being.
